# Supplementary material for: Hybrid Models and Biological Model Reduction with PyDSTool
Source: PLoS Comput Biol. 2012 Aug 9;8(8):e1002628. doi: 10.1371/journal.pcbi.1002628 (PMC3415397; doi:10.1371/journal.pcbi.1002628)
Supplement: Text S4 — Complete source code for the PyDSTool package (version 0.88.120504). Includes API documentation and help files linking to web pages. This file is identical to the current public release on Sourceforge.net. (ZIP) [file pcbi.1002628.s004.zip › PyDSTool/html/PyDSTool.Generator.Radau_ODEsystem'-module.html]

xml version="1.0" encoding="ascii"?


PyDSTool.Generator.Radau\_ODEsystem'


| Home | Trees | Indices | Help | PyDSTool |
| --- | --- | --- | --- | --- |

Package PyDSTool ::
Package Generator ::
Module Radau\_ODEsystem'

[hide private]

[frames] | no frames]

# Module Radau\_ODEsystem'

source code


|  |  |
| --- | --- |
| Classes | [hide private] |

Radau\_ODEsystem  
Wrapper for Radau integrator (with support for
differential-algebraic equations).

radau  
Radau 5 specialization of the basic integrator class.


|  |  |
| --- | --- |
| Functions | [hide private] |

|  |  |
| --- | --- |
| distutil\_destination()  Makes the goofy destination directory string so that we can find where the distutils fortran compiler puts things. | source code |


|  |  |
| --- | --- |
| Variables | [hide private] |

API = `API_class()`

Continuous = `Continuous Domain`

Discrete = `Discrete Domain`

E\_COMPUTFAIL = `0`

E\_NONUNIQUETERM = `10`

Inf = `inf`

LargestInt32 = `2147483647`

NaN = `nan`

W\_BISECTLIMIT = `12`

W\_NONTERMEVENT = `20`

W\_NONTERMSTATEBD = `21`

W\_TERMEVENT = `10`

W\_TERMSTATEBD = `11`

W\_UNCERTVAL = `0`

\_1DimplicitSolveMethods = `['newton', 'bisect', 'steffe']`

\_all\_complex = `(<type 'complex'>, <type 'numpy.complexfloating...`

\_all\_float = `(<type 'float'>, <type 'numpy.floating'>, <type '...`

\_all\_int = `(<type 'int'>, <type 'numpy.integer'>, <type 'numpy...`

\_all\_numpy\_complex = `(<type 'numpy.complex128'>, <type 'numpy....`

\_all\_numpy\_float = `(<type 'numpy.float64'>, <type 'numpy.float...`

\_all\_numpy\_int = `(<type 'numpy.int32'>, <type 'numpy.int32'>, ...`

\_complex\_types = `(<type 'complex'>, <type 'numpy.complexfloati...`

\_float\_types = `(<type 'float'>, <type 'numpy.floating'>)`

\_implicitSolveMethods = `['newton', 'bisect', 'steffe', 'fsolve']`

\_int\_types = `(<type 'int'>, <type 'numpy.integer'>)`

\_num\_equivtype = `{<type 'float'>: <type 'numpy.float64'>, <typ...`

\_num\_maxmin = `{<type 'numpy.int32'>: [-2147483648, 2147483647]...`

\_num\_name2equivtypes = `{'float': (<type 'float'>, <type 'numpy...`

\_num\_name2type = `{'float': <type 'numpy.float64'>, 'int': <typ...`

\_num\_type2name = `{<type 'float'>: 'float', <type 'int'>: 'int'...`

\_num\_types = `(<type 'float'>, <type 'int'>, <type 'numpy.float...`

\_pydstool\_path = `'/Users/robclewley/PyDSTool'`

\_pytypefromtype = `{<type 'numpy.int32'>: <type 'int'>, <type '...`

\_real\_types = `(<type 'int'>, <type 'numpy.integer'>, <type 'fl...`

\_seq\_types = `(<type 'list'>, <type 'tuple'>, <type 'numpy.ndar...`

contained = `contained`

e = `2.71828182846`

errmessages = `{0: 'Computation of trajectory failed', 10: 'Mor...`

errorfields = `{0: ['t', 'error info'], 10: ['t', 'event list']}`

genDB = `Generator internal database class: []`

isfinite = `<ufunc 'isfinite'>`

notcontained = `notcontained`

null\_predicate = `null_predicate_class(None)`

pi = `3.14159265359`

rerr = `<PyDSTool.Redirector.Redirector object at 0x4d185b0>`

rout = `<PyDSTool.Redirector.Redirector object at 0x4d18cb0>`

symbolMapDict = `{'abs': 'fabs', 'mod': 'fmod', 'sign': 'signum'}`

targetLangs = `['c', 'python', 'matlab']`

theGenSpecHelper = `GenSpecHelper()`

uncertain = `uncertain`

warnfields = `{0: ['value', 'interval'], 10: ['t', 'event list'...`

warnmessages = `{0: 'Uncertain value computed', 10: 'Terminal e...`


|  |  |
| --- | --- |
| Variables Details | [hide private] |

### \_all\_complex

Value:
:   ```
    (<type 'complex'>,
     <type 'numpy.complexfloating'>,
     <type 'numpy.complex128'>,
     <type 'numpy.complex64'>,
     <type 'numpy.complex128'>)
    ```

### \_all\_float

Value:
:   ```
    (<type 'float'>,
     <type 'numpy.floating'>,
     <type 'numpy.float64'>,
     <type 'numpy.float32'>,
     <type 'numpy.float64'>)
    ```

### \_all\_int

Value:
:   ```
    (<type 'int'>,
     <type 'numpy.integer'>,
     <type 'numpy.int32'>,
     <type 'numpy.int32'>,
     <type 'numpy.int8'>,
     <type 'numpy.int16'>,
     <type 'numpy.int32'>,
     <type 'numpy.int64'>)
    ```

### \_all\_numpy\_complex

Value:
:   ```
    (<type 'numpy.complex128'>,
     <type 'numpy.complex64'>,
     <type 'numpy.complex128'>)
    ```

### \_all\_numpy\_float

Value:
:   ```
    (<type 'numpy.float64'>,
     <type 'numpy.float32'>,
     <type 'numpy.float64'>)
    ```

### \_all\_numpy\_int

Value:
:   ```
    (<type 'numpy.int32'>,
     <type 'numpy.int32'>,
     <type 'numpy.int8'>,
     <type 'numpy.int16'>,
     <type 'numpy.int32'>,
     <type 'numpy.int64'>)
    ```

### \_complex\_types

Value:
:   ```
    (<type 'complex'>, <type 'numpy.complexfloating'>)
    ```

### \_num\_equivtype

Value:
:   ```
    {<type 'float'>: <type 'numpy.float64'>,
     <type 'int'>: <type 'numpy.int32'>,
     <type 'numpy.integer'>: <type 'numpy.int32'>,
     <type 'numpy.floating'>: <type 'numpy.float64'>,
     <type 'numpy.int8'>: <type 'numpy.int32'>,
     <type 'numpy.int16'>: <type 'numpy.int32'>,
     <type 'numpy.int32'>: <type 'numpy.int32'>,
     <type 'numpy.int32'>: <type 'numpy.int32'>,
    ...
    ```

### \_num\_maxmin

Value:
:   ```
    {<type 'numpy.int32'>: [-2147483648, 2147483647],
     <type 'numpy.float64'>: [-inf, inf]}
    ```

### \_num\_name2equivtypes

Value:
:   ```
    {'float': (<type 'float'>,
               <type 'numpy.floating'>,
               <type 'numpy.float64'>,
               <type 'numpy.float32'>,
               <type 'numpy.float64'>),
     'int': (<type 'int'>,
             <type 'numpy.integer'>,
             <type 'numpy.int32'>,
    ...
    ```

### \_num\_name2type

Value:
:   ```
    {'float': <type 'numpy.float64'>, 'int': <type 'numpy.int32'>}
    ```

### \_num\_type2name

Value:
:   ```
    {<type 'float'>: 'float',
     <type 'int'>: 'int',
     <type 'numpy.integer'>: 'int',
     <type 'numpy.floating'>: 'float',
     <type 'numpy.int8'>: 'int',
     <type 'numpy.int16'>: 'int',
     <type 'numpy.int32'>: 'int',
     <type 'numpy.int32'>: 'int',
    ...
    ```

### \_num\_types

Value:
:   ```
    (<type 'float'>,
     <type 'int'>,
     <type 'numpy.floating'>,
     <type 'numpy.integer'>)
    ```

### \_pytypefromtype

Value:
:   ```
    {<type 'numpy.int32'>: <type 'int'>,
     <type 'numpy.float64'>: <type 'float'>}
    ```

### \_real\_types

Value:
:   ```
    (<type 'int'>,
     <type 'numpy.integer'>,
     <type 'float'>,
     <type 'numpy.floating'>)
    ```

### \_seq\_types

Value:
:   ```
    (<type 'list'>, <type 'tuple'>, <type 'numpy.ndarray'>)
    ```

### errmessages

Value:
:   ```
    {0: 'Computation of trajectory failed',
     10: 'More than one terminal event found'}
    ```

### warnfields

Value:
:   ```
    {0: ['value', 'interval'],
     10: ['t', 'event list'],
     11: ['t', 'var name', 'var value', '\n\tvalue interval'],
     12: ['t', 'event list'],
     20: ['t', 'event list'],
     21: ['t', 'var name', 'var value', '\n\tvalue interval']}
    ```

### warnmessages

Value:
:   ```
    {0: 'Uncertain value computed',
     10: 'Terminal event(s) found',
     11: 'State variable reached bounds (terminal)',
     12: 'Bisection limit reached for event',
     20: 'Non-terminal event(s) found',
     21: 'State or input variable reached bounds (non-terminal)'}
    ```

  


| Home | Trees | Indices | Help | PyDSTool |
| --- | --- | --- | --- | --- |

|  |  |
| --- | --- |
| Generated by Epydoc 3.0.1 on Fri May 4 15:24:03 2012 | http://epydoc.sourceforge.net |
